# RE “The associations between pretreatment neutrophil-to-lymphocyte ratio, sarcopenia and frailty in older patients with head and neck cancer”

**DOI:** 10.1016/j.jarlif.2026.100066

**Published:** 2026-03-05

**Authors:** Erkan Topkan, Efsun Somay, Sibel Bascil, Ugur Selek

**Affiliations:** a Department of Oral and Maxillofacial Surgery, Faculty of Dentistry, Baskent University, Ankara, Turkey; b Department of Radiation Oncology, Faculty of Medicine, Baskent University, Adana, Turkey; c Department of Periodontology, Faculty of Dentistry, Baskent University, Ankara, Turkey; d Department of Radiation Oncology, School of Medicine, Koc University, Istanbul, Turkey

**Keywords:** Frailty, Sarcopenia, Neutrophil-to-lymphocyte ratio, Head and neck cancer, Geriatric assessment, Inflammation

Dear Editor,

We congratulate Meerkerk and colleagues on their investigation of the relationships between pretreatment neutrophil-to-lymphocyte ratio (NLR), sarcopenia, and frailty in older patients with head and neck cancer (HNC)[1]. The authors deserve commendation for defining sarcopenia in accordance with the European Working Group on Sarcopenia in Older People criteria[2], requiring concurrent reductions in skeletal muscle strength and mass—an approach that remains uncommon in oncologic research, where imaging-only surrogates are frequently employed. Notwithstanding this definitional rigor, two aspects warrant further consideration, as they may influence the study's clinical applicability and interpretation.

First, although the authors report significant associations between elevated NLR and frailty[1], the discriminatory performance of NLR is inadequate for screening purposes; with a specificity of only 47 % and an overall accuracy of 61 %, nearly half of non-frail patients would be misclassified as potentially frail[3]. Such performance would substantially increase unnecessary referrals for comprehensive geriatric assessment (GA) in real-world practice, contradicting the stated objective of improving efficiency in resource-limited settings. Screening instruments should therefore be evaluated primarily on clinical utility and operational performance, rather than on statistical association alone. Established tools, such as the G8, demonstrate a more favorable balance of sensitivity and specificity and have undergone prospective validation across diverse oncologic populations[4]. When evaluated against this benchmark, NLR does not confer meaningful incremental value.

Second, the study underscores a persistent conceptual issue in the frailty literature: the tendency to conflate frailty with biological surrogates of inflammation or muscle mass. As demonstrated in the multivariable analyses by Meerkerk and colleagues, comorbidity burden and nutritional impairment largely account for the observed association between NLR and frailty[1]. This finding suggests that NLR may function primarily as a nonspecific marker of global disease severity or catabolic stress, rather than reflecting a frailty-specific biological mechanism. Frailty is a multidimensional functional syndrome characterized by impaired physiologic reserve across multiple systems[5]. Reducing this construct to an inflammatory ratio risks biological oversimplification and interpretive overstatement. This concern is particularly salient given that structured GA domains already capture vulnerability more directly and demonstrate greater prognostic relevance.

In conclusion, although the available data do not support the use of NLR as a reliable frailty screening tool, Meerkerk and colleagues demonstrated that systemic inflammation, sarcopenia, and frailty frequently coexist in older patients with HNC[1]. Future investigations would benefit from moving beyond isolated biomarkers toward integrative, prospectively validated models that preserve the functional and multidimensional nature of frailty.

## Authors’ contributions

The author conceptualized the commentary, performed the critical analysis of the published study, and wrote the manuscript.

## Ethics approval and consent to participate

Not applicable. This Letter to the Editor does not involve original data collection, human participants, or animal experimentation.

## Consent for publication

Not applicable.

## Availability of data and materials

Not applicable.

## Funding

No specific funding was received for this work.

## Declaration of the use of generative AI and AI-assisted technologies in scientific writing and in figures, images, and artwork

Not applicable.

## CRediT authorship contribution statement

**Erkan Topkan:** Writing – review & editing, Writing – original draft, Visualization, Validation, Supervision, Software, Resources, Project administration, Methodology, Investigation, Formal analysis, Data curation, Conceptualization. **Efsun Somay:** Writing – review & editing, Writing – original draft, Visualization, Validation, Supervision, Software, Resources, Project administration, Methodology, Investigation, Formal analysis, Data curation, Conceptualization. **Sibel Bascil:** Writing – review & editing, Writing – original draft, Visualization, Validation, Supervision, Software, Resources, Project administration, Methodology, Investigation, Formal analysis, Data curation, Conceptualization. **Ugur Selek:** Writing – review & editing, Writing – original draft, Visualization, Validation, Supervision, Software, Resources, Project administration, Methodology, Investigation, Formal analysis, Data curation, Conceptualization.

## Declaration of competing interest

The authors declare that they have no known competing financial interests or personal relationships that could have appeared to influence the work reported in this paper.

The author is an Editorial Board Member/Editor-in-Chief/Associate Editor/Guest Editor for *[Journal name]* and was not involved in the editorial review or the decision to publish this article.

## References

[1] Meerkerk C.D.A., Emmelot-Vonk M.H., Haitjema S., de Bree R. (2025). The associations between pretreatment neutrophil-to-lymphocyte ratio, sarcopenia and frailty in older patients with head and neck cancer. J Aging Res Lifestyle.

[2] Cruz-Jentoft A.J., Bahat G., Bauer J. (2019). Sarcopenia: revised European consensus on definition and diagnosis. Age Ageing.

[3] Decoster L., Van Puyvelde K., Mohile S. (2015). Screening tools for multidimensional health problems warranting a geriatric assessment in older cancer patients: an update on SIOG recommendations. Ann Oncol.

[4] Bruijnen C.P., Heijmer A., van Harten-Krouwel D.G. (2021). Validation of the G8 screening tool in older patients with cancer considered for surgical treatment. J Geriatr Oncol.

[5] Zwart A.T., van der Hoorn A., van Ooijen P.M.A. (2019). CT-measured skeletal muscle mass used to assess frailty in patients with head and neck cancer. J Cachexia Sarcopenia Muscle.

